# Reliability of dried blood spot (DBS) cards in antibody measurement: A systematic review

**DOI:** 10.1371/journal.pone.0248218

**Published:** 2021-03-15

**Authors:** Fahimah Amini, Erick Auma, Yingfen Hsia, Sam Bilton, Tom Hall, Laxmee Ramkhelawon, Paul T. Heath, Kirsty Le Doare

**Affiliations:** 1 Paediatric Infectious Disease Research Group, Institute for Infection and Immunity, St. George’s University of London, London, United Kingdom; 2 Department of Biology, University of Lyon, Université Claude Bernard Lyon, ENS de Lyon, CNRS, UMR, Lyon, France; 3 School of Pharmacy, Queen’s University Belfast, Belfast, United Kingdom; 4 St Georges University Hospitals NHS Foundation Trust, Tooting, London, United Kingdom; 5 Pathogen Immunology Group, Public Health England, Porton Down, United Kingdom; University of Liverpool Institute of Infection and Global Health, UNITED KINGDOM

## Abstract

**Background:**

Increasingly, vaccine efficacy studies are being recommended in low-and-middle-income countries (LMIC), yet often facilities are unavailable to take and store infant blood samples correctly. Dried blood spots (DBS), are useful for collecting blood from infants for diagnostic purposes, especially in low-income settings, as the amount of blood required is miniscule and no refrigeration is required. Little is known about their utility for antibody studies in children. This systematic review aims to investigate the correlation of antibody concentrations against infectious diseases in DBS in comparison to serum or plasma samples that might inform their use in vaccine clinical trials.

**Methods and findings:**

We searched MEDLINE, Embase and the Cochrane library for relevant studies between January 1990 to October 2020 with no language restriction, using PRISMA guidelines, investigating the correlation between antibody concentrations in DBS and serum or plasma samples, and the effect of storage temperature on DBS diagnostic performance.

We included 40 studies in this systematic review. The antibody concentration in DBS and serum/plasma samples reported a good pooled correlation, (*r*^*2*^ = 0.86 (ranged 0.43 to 1.00)). Ten studies described a decline of antibody after 28 days at room temperature compared to optimal storage at -20°C, where antibodies were stable for up to 200 days. There were only five studies of anti-bacterial antibodies.

**Conclusions:**

There is a good correlation between antibody concentrations in DBS and serum/plasma samples, supporting the wider use of DBS in vaccine and sero-epidemiological studies, but there is limited data on anti-bacterial antibodies. The correct storage of DBS is critical and may be a consideration for longer term storage.

## Introduction

Infectious diseases are a major global cause of morbidity and mortality affecting all age groups, especially young infants. Many of these diseases could be prevented through vaccination. However, vaccine clinical trials require blood draws from infants, which are often difficult because of both the volume required and the need for correct handling and storage of the sample [1]. This is especially true in low-income countries because of issues with cold chain maintenance and logistics of transportation from remote locations to a centralised research laboratory for processing. Detection and quantification of antibodies in the serum/plasma, offer a rapid and accurate assessment of vaccine responses. To use serum/plasma samples for serological tests, trained healthcare professionals are required to draw blood [2,3]. As well as a trained professional, there is a need for specific equipment such as vacutainers to collect whole blood and a centrifuge to separate serum/plasma from whole blood. Aside from specialised equipment, freezers are required to store the samples optimally prior to analysis. Due to the expensive nature of handling (i.e. storage, electricity) of blood samples, vaccine clinical trials are often problematic in a resource-limited setting [1]. Dried blood spots (DBS) would be a possible alternative, as they require a less complex procedure to collect than whole blood, especially from young infants [3,4].

The use of DBS as a diagnostic tool dates back to the 19th century, pioneered by Robert Guthrie for neonatal metabolic disorder screening [5]. In addition to screening for metabolic disorders, DBS cards have been utilised for human immunodeficiency virus (HIV) screening, laboratory quality control, drug testing and detection of pathogens in diverse sample types, including blood and dried plasma spots [6,7]. Numerous studies have also demonstrated that antibodies can be detected on DBS, such as in the prospective cohort study of congenital cytomegalovirus [8] and HIV infection [9]. DBS samples are cost-effective as easily portable equipment (i.e. lancet device and Whatmann 909 paper) can be used and does not require any specialist training.

Regardless of the broad use of DBS in a wide range of immunological bioanalyses, sensitivity and specificity remains uncertain regarding antibody quantification. There are no approved regulations or manufacturers’ guideline on assay protocols for quantifying antibody concentration in DBS. There are also differences in DBS in terms of the cards themselves including the size and thickness of the spots and the material used to manufacture the cards. Further, there are no guidelines on how analysis should be conducted, including optimal elution methods.

This systematic review aims to assess the evidence for the use of DBS to accurately measure antibody concentrations from natural exposure and vaccination. Further, we review long-term DBS storage conditions in preparation for future sero-epidemiological or vaccine studies.

## Methodology

The protocol used for this review is registered with PROSPERO [CRD42019127840].

### Search strategy

PRISMA was used as a guideline to conduct this systematic review [10]. We searched the electronic databases Embase, Medline and Cochrane library for studies published between January 1^st^, 1990 and October 15^th^ 2020, comparing antibody levels in serum/plasma and DBS obtained from individuals below the age of 80 years. We additionally searched for articles describing stability of DBS at different storage temperatures over time. No language restriction was applied. The search strategy used a combination of MeSH and free terms for ‘dried blood spot’ OR ‘Guthrie card’ AND ‘antibody’. The database was last searched on the 15^th^ October 2020.

The PRISMA checklist and the full search string are available in the S2 Method.

### Eligibility criteria

The studies that were considered eligible for inclusion were original research articles, concerning infectious diseases in humans, comparing antibody titres/concentrations in serum/plasma to DBS or describing stability of antibodies in DBS from longitudinal studies of storage at different temperatures. We included studies from all countries. Opinion pieces, reviews, comments, letters and conference abstracts were excluded. Studies that used animals to compare antibody levels in serum/plasma and DBS were also excluded. Studies that had insufficient data (absence of two or more of the following: number of participants, age, sensitivity, specificity, correlation of antibody levels in matched DBS-serum/plasma samples) were also excluded. Additionally, we included all studies that investigated the stability of antibodies in DBS samples.

### Study selection

Two independent reviewers (FA and EA) screened the titles and abstracts of the identified studies. After the initial screening, the reviewers obtained full texts of reports and they independently reviewed each article to determine whether it would be included in the final review. Disagreement on studies were resolved in discussion with a third reviewer (KLD).

### Data extraction

The reviewers (FA and EA) independently extracted the data from the included studies using PICO (patient, intervention, comparison and outcome) [11]. The following information was extracted from the selected studies: author, publication year, country, journal, infectious disease, aim of research, study design, duration of study, number of participants, mean age, sensitivity, specificity, method of sample collection, method of sample storage, laboratory tests used for confirmation, elution method and outcome. The country income of the included studies was classified by using their respective gross domestic product (GDP) using the World Bank [12]. All studies were either classified as a low-middle income country (LMIC) (which consisted of low-income, lower middle-income and upper middle-income countries) or as a high-income country (HIC).

### Data synthesis and analysis

Due to the high heterogeneity of study design, participants and outcomes, we were only able to conduct a narrative synthesis of included studies, summarising the findings with respect to each infectious disease. We calculated the pooled estimates of specificity, sensitivity and correlation coefficient using the accuracy data (true positive, true negative, false positive and false negative).

### Risk of bias

The risk of bias was assessed by FA using the Cochrane Risk of Bias for non-randomised studies (ROBINS-I) tool [13]. This included information on bias due to confounding, bias in selection of participants into the study, bias in classification of interventions, bias due to deviations from intended interventions, bias due to missing data, bias in measurement of outcomes and bias in selection of the reported results. Due to the nature of the interventions considered in this review, the study’s participants could not be blinded.

## Results

**Fig 1:**
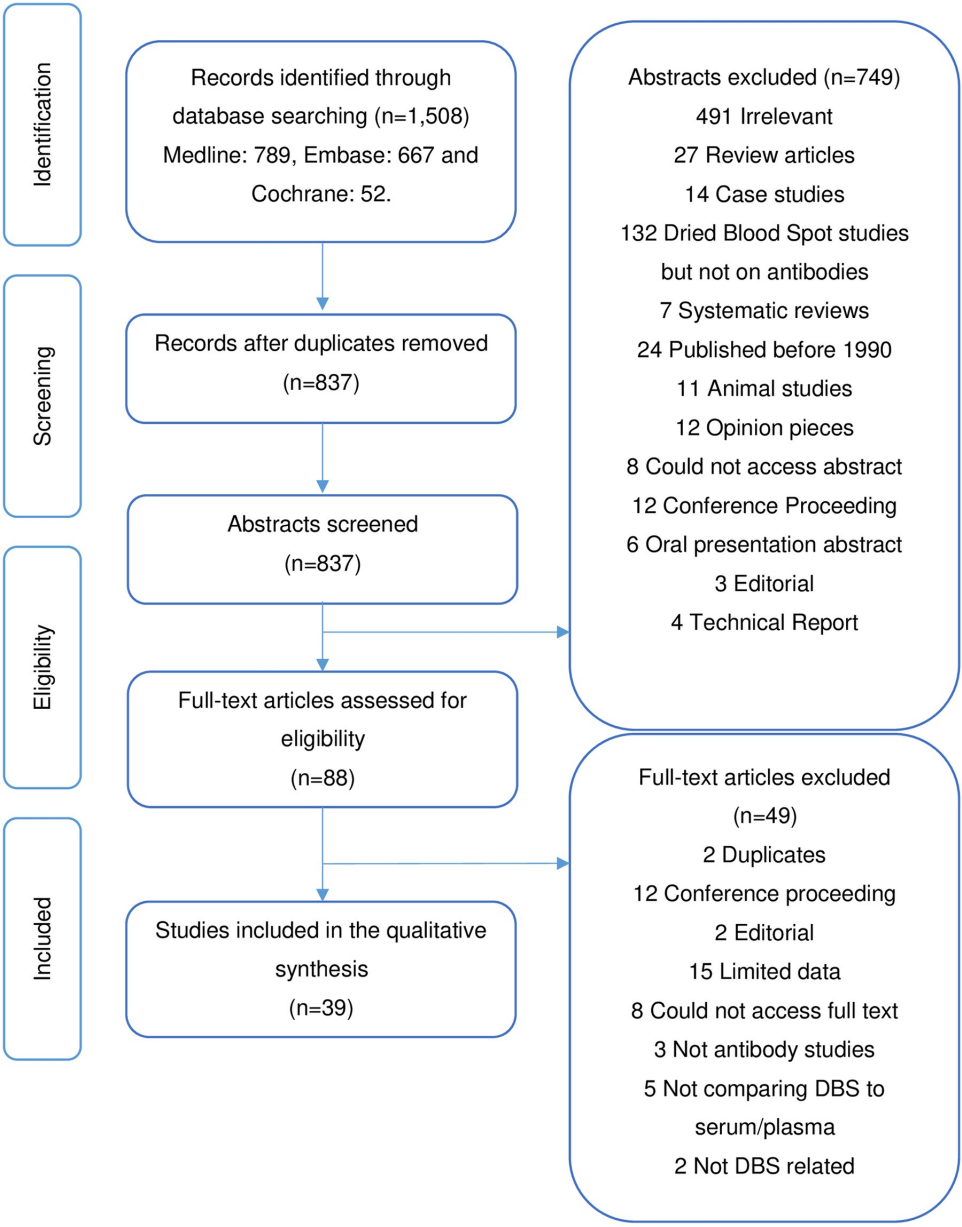
Prisma flowchart. Flowchart of studies included in the systematic review on detecting antibodies from DBS compared to venous blood samples (plasma/serum).

We identified 1,508 studies from the electronic databases published between 1^st^ January 1990 to 15^th^ October 2020. Using our search term, we sourced 789 papers from Medline, 667 from Embase and 52 from Cochrane. After the removal of duplicates, 837 studies were identified for abstract screening. A total of eighty-eight full text studies were assessed for eligibility, forty studies met the criteria for inclusion (Fig 1). An additional five papers investigating only antibody stability were also included.

### Study characteristics

The characteristics of the included studies are summarised in Table 1 and the antibody assessment is summarised in Table 2. Overall, DBS and serum samples from 16,255 individuals were included: 13,742 (84.5%) adults, 560 (3.4%) 5- to 17-year old’s and 2,082 (12.8%) less than five years old. Two studies reported antibodies against hepatitis A [14,15], nine hepatitis B [16–18,26–28,46,48,51], ten hepatitis C [19–22,26–28,37,46,48], eight HIV [23–28,46,50], three *human papillomavirus* (HPV) [29,30,37], three measles [31,32,42], three rubella [33,34,42], two syphilis [40,46], two *H*. *pylori* [35,47] and two malaria [38,49]. Twelve papers reported on Chagas disease [35], Epstein-Barr virus [36], HPV, *H*. *pylori*, hepatitis C and polyomavirus [37], Strongyloidiasis [39], pertussis [41], hepatitis E [56], *Vibrio cholera* [2], measles, mumps and rubella [42], tuberculosis and cytomegalovirus [43], toxoplasmosis [44], trypanosoma [45], Covid-19 [52], respectively.

**Table 1:** Characteristics of the included studies.

AUTHOR, YEAR	COUNTRY	GDP	STUDY PERIOD	STUDY DESIGN	SAMPLE SIZE	AGE	DISEASE	CONCLUSION
**GIL A, 1997** [14]	Spain	HIC	Nr	Cross-sectional	298	15.3 ± 1.2 years	HAV	Anti-HAV antibodies are stable on DBS and ELISA is a good assay to determine anti-HAV antibodies
**MELGACO J, 2011** [15]	Brazil	LMIC	March 2007	Cross-sectional	74	20.6 ± 1.9 years	HAV	There is a strong correlation in anti-HAV antibodies in plasma and DBS; suggesting that commercial assays can be used to detect anti-HAV antibodies in DBS, without the need to recalculating cut-off values
**FLORES L, 2018** [16]	Brazil	LMIC	Nr	Cross-sectional	155	Adults (18 and above)	HBV	Amongst HIV positive and negative subjects, DBS could be used to quantify and detect anti-HBs
**VILLAR M, 2011** [17]	Brazil	LMIC	183 days	Cross-sectional	522	Adults (18 and above)	HBV	Good correlation of HBV markers in serum and DBS Until 63 days, HBV markers could be detected in various temperatures, but storing DBS at -20C yielded consistent results
**MOHAMED S, 2013** [18]	France	HIC	Nr	Cohort	230	Adults (18 and above)	HBV	DBS is an alternative reliable sample to plasma specimens to quantify and detect Hepatitis B antigens
**DOKUBO K, 2014** [19]	USA	HIC	July 2010- June 2011	Cross-sectional	148	Adults (18–30 years)	HCV	DBS and plasma showed a good correlation for anti-HCV detection
**TEJADA-STROP A, 2015** [20]	USA	HIC	Samples collected 5 years prior to testing	Retrospective Case-control	33	Adults (18 and above)	HCV	The sensitivity of anti-HCV IgG detection in stored DBS was corresponding to the matched plasma sample.
**TUAILLON E, 2010** [21]	France	HIC	Nr	Case-control	200	Adults (18 and above)	HCV	DBS is reliable as serum specimens and it can be used as an alternative to detect anti-HCV
**BRANDAO C, 2013** [22]	Brazil	LMIC	January 2009- March 2010	Cross-sectional	386	21–60 years	HCV	DBS is a good alternative to detect anti-HCV, although it was necessary to increase DBS sample volume due to low amount of antibody levels as compared to sera samples
**SARGE-NIJE R, 2006** [23]	Gambia	LMIC	Nr	Cross-sectional	200	Adults (18 and above)	HIV	HIV test results from eluted filter paper specimen are comparable to those obtained from corresponding serum samples, depending on the strategy used.
**CASTRO A, 2008** [24]	Brazil	LMIC	2005–2006	Cross-sectional	457	Ranged from 2 months to 78 years	HIV	DBS has a high-performance characteristic when compared to serum specimens
**BOILLOT F, 1997** [25]	Sierra Leone	LMIC	April 1994	Cross-sectional	359	Adults (18 and above)	HIV	DBS is feasible to perform HIV sero-surveillance in a national tuberculosis control program
**KANIA D, 2013** [26]	Burkina Faso	LMIC	21st June- 1st July 2011	Cross-sectional	218	29.8 ± 11.0 years	HBV, HCV and HIV	There was good analytical performance of DBS assay obtained in this study for HCV and HBV The ODs of DBS from HIV, HCV or HBV positive subjects did not overlap with ODs of DBS samples from negative individuals
**ROSS S, 2013** [27]	Germany	HIC	Nr	Cross-sectional	726	Adults (18 and above)	HBV, HCV and HIV	There is a good correlation of antibody levels across the three diseases in matched serum and DBS.
**LEE C, 2011** [28]	Malaysia	LMIC	November 2009- March 2010	Cross-sectional	600	Adults (18 and above)	HBV, HCV and HIV	DBS is as reliable as serum, although different cut-off points need to be used to validate test positivity in DBS
**BHATIA R, 2019** [29]	UK	HIC	Nr	Cross-sectional	96	Females (21–59 years)	HPV	A strong correlation between anti-HPV16L1 IgG and IgA was observed in serum and DBS.
**LOUIE K, 2018** [30]	UK	HIC	Nr	Cohort	Vaccinated: 50 Unvaccinated:103	Vaccinated females: 21 years (median) Unvaccinated females: 26 years (median)	HPV	Although sensitivity levels of DBS are slightly lower than serum; DBS is an appropriate alternative to serum for HPV vaccine surveillance
**UZICANIN A, 2011** [31]	Uganda	LMIC	Nr	Cross-sectional	Measles: 274 Non-measles: 75	Measles: 31 ± 25.8 (mean months ±SD) Non-measles: 41 ± 29.5 (mean months ± SD)	Measles	Although the non-measles group had a low concordance group; the overall findings indicate that DBS are a feasible alternative to serum for serological confirmation
**COLSON K, 2015** [32]	Mexico and Republic of Nicaragua	LMIC	2015	Cross-sectional	Mexico:1134 Nicaragua:454	Mexico- 17.5 ± 0.19 (months mean ± SE) Nicaragua- 17.7 ± 0.23 (months mean ± SE)	Measles	It is feasible to conduct sero-surveys using DBS samples in low-resource settings, as the antibody levels are highly consistent in matched serum and DBS samples
**PUNNARUGSA V, 1991** [33]	Thailand	LMIC	Nr	Cross-sectional	1000	Adults (18 and above)	Rubella	80.7% of DBS had similar antibody titres to matched serum.
**HELFAND F, 2007** [34]	Peru	LMIC	June 2004- May 2005	Cross-sectional	376	Children (8 months and above)	Rubella	There was a good correlation between serum and DBS; which suggests that DBS is an acceptable to be used as a sample for serological tests
**HOLGUIN A, 2013** [35]	Spain	HIC	May 2011- March 2012	Cross-sectional	147	Adults (18 and above)	Chagas	With a cut-off point of ≥0.88, there are low numbers of false-negative DBS
**EICK G, 2017** [36]	USA	HIC	November 2014- February 2015	Cohort	208	Adults (18–77 years)	Epstein-Barr virus	This study showed that there is a high correlation of Epstein-Barr virus antibodies in plasma and DBS.
**WATERBOER T, 2012** [37]	Mongolia	LMIC	Nr	Cross-sectional	985	Females (16–63 years)	HPV, *H*. *pylori*, HCV and Polyomavirus	There was a good correlation between serum and DBS in high-titre antibodies when compared to low-titre antibodies
**DUARTE E, 2002** [38]	Brazil	LMIC	September 1996	Retrospective	210	1–64 years (25.3 years)	Malaria	DBS is as reliable as serum samples to determine malaria status in patients
**FORMENTI F, 2016** [39]	Ecuador	LMIC	Nr	Cross-sectional	235 (174 children and 61 adults)	Adults (19–70 years) Children (7–16 years)	Strongyloidiasis	Good concordance between standard serology and DBS results
**SMIT P, 2013** [40]	Tanzania	LMIC	Nr	Cohort	1645	15–84 years (31.9 years)	Syphilis	It is recommended that Treponema pallidum particle agglutination assay is used to quantify antibody levels in DBS
**VAN OMMEN C, 2012** [41]	Netherlands	HIC	August 2006- April 2007	Cross-sectional	129	Mothers and new-born’s	Pertussis	Anti-PT IgG from cord blood in DBS and serum showed a high coefficient of correlation
**CONDORELLI F, 1994** [42]	Italy	HIC	January 1993- March 1993	Cross-sectional	228	Children and adults	Measles, mumps and rubella	(1) Antibodies are stable for at least for 2 weeks at room temperature (2) The antibody levels DBS and serum showed a good agreement as they were higher than 96%
**AABYE M, 2012** [43]	Finland	HIC	Nr	Cross-sectional	52	Adults (18 and above)	Tuberculosis and Cytomegalovirus	Correlation of IP-10 marker was excellent in DBS and plasma samples
**HEGAZY M, 2020** [44]	Egypt	LMIC	Nr	Cross-sectional	101	10–70 years	Toxoplasmosis	DBS can be used to test for *Toxoplasma* as it has high diagnostic accuracy
**GEERTS M, 2020** [45]	Democratic Republic of Congo	LMIC	Nr	Cross-sectional	132	Nr	Trypanosoma brucei	The high sensitivity and specificity suggests that DBS can be used determine *Trypanosoma brucei* infection status
**MA J, 2020** [46]	China	HIC	Nr	Cross-sectional	429	Adults (18 and above)	HIV, Hepatitis C and syphilis	The antibody concentration in DBS samples were comparable to the antibody concentration in the matched plasma samples
**KUMAR A, 2019** [47]	India	LMIC	January 2018- May 2018	Observational	88	Adults (18 and above)	*H*. *pylori*	No difference in antibody levels were seen in matched DBS and plasma samples
**VILLAR L, 2020** [48]	Argentina	HIC	October 2014- December 2014	Cross-sectional	622	Adults (36.6±14.3)	HBV and HCV	Although the sensitivity % for anti-HBc, DBS samples still can be utilised for detecting anti-HBV and anti-HCV antibody concentrations
**ROSAS-AGUIRRE A, 2020** [49]	Peru	HIC	August 2012-September 2012	Longitudinal cohort	470	Adults (18 and above)	*Plasmodium vivax*	To detect anti-P.vivax antibodies, it is better to use serum rather than DBS samples
**STEFIC K, 2019** [50]	France	HIC	Nr	Cross-sectional	10	Adults (18 and above)	HIV	This study is further showing evidence that DBS samples are also suitable for detecting HIV antibodies, as well as, for use in population surveillance
**CRUZ H, 2020** [51]	Brazil	LMIC	2009–2014	Cross-sectional	2309	Adults (18 and above)	HBV	HBsAg in DBS samples had the best sensitivity percentage in this study. This study showed that DBS samples can be utilised for detecting HBV infections
**MORLEY G, 2020** [52]	UK	HIC	2020	Cross-sectional	87	Adults (18 and above)	Coronavirus	The results of this study illustrate that antibodies in DBS samples are highly comparable to serum samples

Abbreviations: ^a^ GDP: Gross domestic product; ^b^ HIC: High Income Country; ^c^ LMIC: Lower Middle-Income Countries; ^d^ HAV: Hepatitis A virus; ^e^ HBV: Hepatitis B virus; ^f^ HCV: Hepatitis C virus; ^g^ HIV: Human Immunodeficiency virus; ^h^ HPV: Human papillomavirus; ^i^ anti-HBs: Hepatitis B surface antibody; ^j^ Nr: Not reported.

**Table 2:** Immunological assessment of the included studies.

AUTHOR, YEAR	TYPE OF SCREENING	LABORATORY TEST USED TO CONFIRM PATHOGEN	ELUTION METHOD OF DBS	SAMPLE STORAGE
**GIL A, 1997** [14]	DBS and serum	ELISA	DBS were eluted for 12 hours at room temperature in 1 ml of 0.115% saline/1.5% bovine albumin	DBS: 4°C; Serum: -20°C
**MELGACO J, 2011** [15]	DBS and plasma	ELISA	12.5mm-diameter DBS were eluted at 4°C overnight in 350 μl PBS/0.2% Tween-20/5% bovine plasma albumin	-20°C
**FLORES L, 2018** [16]	DBS and serum	ELISA	Nr	Nr
**VILLAR M, 2011** [17]	DBS and serum	ELISA	6-mm DBS samples were eluted in 700 μl of PBS/0.5% BSA for HBsAg and anti-HBc antibody quantification. 12.5mm DBS samples were eluted in 300 μl of PBS/0.5% BSA for HBsAg and anti-HBc antibody quantification. All DBS samples were incubated at 2°-6°C for 18–24 hours.	-20°C
**MOHAMED S, 2013** [18]	DBS and plasma	CIA	12-mm diameter DBS were eluted at room temperature in continued agitation in 450 μl PBS	DBS: RMT; Serum: Nr
**DOKUBO K, 2014** [19]	DBS and serum	ELISA	6.35-mm diameter DBS were eluted overnight at 2°-8°C in 125 μl PBS	-70°C
**TEJADA-STROP A, 2015** [20]	DBS and plasma	CIA and ELISA	12-mm diameter DBS were eluted overnight at 4°C in 500 μl PBS/Tween	-20°C for 5 years
**TUAILLON E, 2010** [21]	DBS and serum	ELISA	6-mm diameter DBS were eluted on shaker overnight in 200 μl ELISA kit sample diluent	DBS: -20°C; Serum: Nr
**BRANDAO C, 2013** [22]	DBS and serum	ELISA	3-mm diameter DBS were eluted for 18–24 hours at 4°-8°C in 300 μl PBS/0.5% BSA	-20°C
**SARGE-NIJE R, 2006** [23]	DBS and serum	ELISA	5.5-mm diameter DBS eluted overnight at 4°C in 100 μl or 200 μl PBS/Tween-20/0.005% Sodium azide	Nr
**CASTRO A, 2008** [24]	DBS and serum	ELISA	4.7-mm diameter DBS eluted in ELISA kit sample diluent	DBS: -20°C; Serum: Nr
**BOILLOT F, 1997** [25]	DBS and serum	ELISA	DBS in PBS was placed on rotatory shaker for 90 mins, followed by overnight incubation at 4°C	-20°C
**KANIA D, 2013** [26]	DBS and plasma	ELISA	6-mm diameter DBS were eluted overnight in 300 μl (HBsAg and HCV) or 150 μl (anti-HBc) PBS	Nr
**ROSS S, 2013** [27]	DBS and serum	Architect system	DBS were eluted overnight at room temperature on a shaker in 1 ml PBS/0.05% Tween-20/0.08% Sodium azide	Nr
**LEE C, 2011** [28]	DBS and plasma	Nr	5.5-mm diameter DBS were eluted for 1 hour at 4°C in 500 μl miliQ water	DBS: -20°C; Serum: -80°C
**BHATIA R, 2019** [29]	DBS and serum	ELISA	DBS were eluted in 800 μl PBS/0.05% Tween-20	DBS: 4°C; Serum: Nr
**LOUIE K, 2018** [30]	DBS and serum	ELISA	9x 2-mm diameter DBS were eluted overnight at 4°C on a shaker in 200 μl PBS/0.05% Tween-20	4°C
**UZICANIN A, 2011** [31]	DBS and serum	ELISA	6.35-mm diameter DBS were eluted in 250 μl PBS/Tween-20/5% non-fat dry milk followed by placing it on a plate shaker for 30 mins. Following on, the samples were incubated for 16 hours at 4°C	-20°C
**COLSON K, 2015** [32]	DBS and serum	ELISA	6-mm diameter DBS were eluted for 14–16 hours at 6°-8°C in 400 μl PBS	-20°C
**PUNNARUGSA V, 1991** [33]	DBS and serum	Unclear	Unclear	DBS: RMT; Serum: -20°C
**HELFAND F, 2007** [34]	DBS and serum	ELISA	Nr	-20°C
**HOLGUIN A, 2013** [35]	DBS and serum	Architect Chagas assay	2x 1.1-mm diameter DBS were eluted overnight at room temperature on a shaker in 300 μl PBS	-80°C
**EICK G, 2017** [36]	DBS and plasma	ELISA	3.2-mm diameter DBS were eluted overnight at 4°C on a shaker in 250 μl ELISA kit sample diluent	-80°C
**WATERBOER T, 2012** [37]	DBS and serum	Multiplex serology (Luminex 100 analyser)	2.85-mm diameter DBS were eluted overnight at 4°C in 100 μl PBS	-20°C
**DUARTE E, 2002** [38]	DBS and serum	ELISA	Unclear	DBS: -5°C to -10°C Serum: -70°C
**FORMENTI F, 2016** [39]	DBS and serum	ELISA	8 DBS were eluted overnight at room temperature in PBS/Tween-20	-20°C
**SMIT P, 2013** [40]	DBS and plasma	TPPA, ELISA and TPHA	6-mm diameter DBS were eluted overnight at 4°C in 100 μl PBS/0.05% Tween-80	-20°C
**VAN OMMEN C, 2012** [41]	DBS and serum	ELISA	2x 3.18-mm diameter DBS were vortexed at room temperature for 1 hour in 400 μl 0.5%BSA/0.01% Tween-20	-20°C
**CONDORELLI F, 1994** [42]	DBS and serum	ELISA	DBS were eluted at room temperature for 30 mins in agitation in 50 μl 0.05% PBS/Tween-20	DBS: 4°C; Serum: -20°C
**AABYE M, 2012** [43]	DBS and plasma	ELISA	Unclear	-80°C
**HEGAZY M, 2020** [44]	DBS and serum	ELISA	DBS were eluted in 0.2mL PBS at room temperature overnight on an automatic shaker. Samples were then centrifuged at 1000xg for 10 minutes	-20°C
**GEERTS M, 2020** [45]	DBS and plasma	ELISA	6-mm DBS spots were eluted overnight in 400 μl *g*-iELISA sample diluent	Nr
**MA J, 2020** [46]	DBS and plasma	ELISA	DBS samples were eluted for comparison for all three antibodies in 1%Tween-20/PBS, 1%TritonX100/PBS, 1%Tween-20 and 1% TritonX100. Furthermore, the elution effect of Tween-20/PBS buffer was compared at 1% and at 3%	DBS: -20°C; Plasma: -20°C
**KUMAR A, 2019** [47]	DBS and plasma	ELISA	6-mm DBS spot was eluted in 500 μl of diluent buffer (part of the ELISA kit) for 2–3 hours at room temperature on a plate shaker	DBS: -20°C; Plasma: -80°C
**VILLAR L, 2020** [48]	DBS and serum	PCR	For anti-HCV, a 3-mm spot was placed into a microtube containing 300 μl of PBS/0.5%BSA at 4–8°C for 18–24 hours. A 6-mm spot was eluted in 700 μl of PBS/0.5%BSA at 4–8°C for 18–24 hours for HBsAg and anti-HBc detection.	-20°C
**ROSAS-AGUIRRE A, 2020** [49]	DBS and serum	ELISA	5-mm disc was eluted overnight at 4°C in PBS/non-fat milk/0.05%Tween-20	DBS: 4°C; Serum: -70°C
**STEFIC K, 2019** [50]	DBS and plasma	ELISA	6-mm spot was placed in 150 μl 0.01M-PBS/10%BSA/0.05%Tween-20 and it was incubated overnight at 4°C	Nr
**CRUZ H, 2020** [51]	DBS and serum	ELISA	To detect HBsAg and total anti-HBc, a 6-mm DBS spot was eluted in 700 μl PBS/0.05%BSA. For anti-HBc detection, a 12.5 mm spot was eluted in 300 μl of PBS/0.05%BSA.	-20°C
**MORLEY G, 2020** [52]	DBS and serum	ELISA	One DBS spot was eluted in 250 μl of 0.05% PBS/Tween-20. The tubes were briefly vortexed before it was incubated overnight at toon temperature. On the following day the tubes were centrifuged for 10 minutes at 10,600 xg at room temperature.	Nr

Abbreviations: ^a^ HAV: Hepatitis A virus; ^b^ HCV: Hepatitis C virus; ^c^ HIV: Human Immunodeficiency virus; ^d^ HPV: Human papillomavirus; ^e^ HBsAg: Hepatitis B surface antigen; ^f^ anti-HBc: Hepatitis B core antibody; ^g^ anti-HBs: Hepatitis B surface antibody; ^h^ BSA: Bovine serum albumin; ^i^ CIA: Chemiluminescence immunoassay; ^i^ PBS: Phosphate buffered saline; ^j^ PCR: Polymerase chain reaction; ^k^TPPA: Treponema pallidum particle agglutination assay; ^l^ TPHA: Treponema pallidum hemagglutination assay; ^m^ Nr: Not reported; ^n^ RMT: Room temperature.

Twelve studies were conducted in Europe [14,18,21,27,29,30,35,41–43,50,52], four in North America [19,20,32,36], eleven in South America [15–17,22,24,34,38,39,48,49,51], seven in Africa [23,25,26,31,40,44,45] and five in Asia [28,33,37,46,47].

The risk of confounding was high in all included studies as the risks for measure of outcomes, missing data and deviation from intended interventions were unclear (S1 Table). Twenty-two of the studies had a moderate risk of bias for reporting [15,17–19,22,24,27–32,35,37,39,40,44,46–48,51,52], whereas, seventeen of the studies had a high risk of bias for reporting [14,16,20,21,23,25,26,33,34,36,38,41–43,45,49,50]. All studies had an overall high risk of bias (S1 Table). Blinding the laboratory personnel to the results of tests were not reported in any of included studies. Thirteen studies reported the stability of DBS, 35 out of the 39 studies reported the elution method and 33 out of the 39 studies reported the diagnostic performance.

### Elution method

Thirty-two (89%) reported the methodology used to elute the DBS samples [14,15,17–32,35–37,39–42,44–52]. The shortest incubation period of the DBS in elution buffer (50 μl 0.05% PBS/Tween-20) was 30 minutes [42] and the longest incubation period of the DBS in elution buffer (700 μl and 300 μl PBS/0.05% BSA) was 18–24 hours [17]) (Table 2). Nine studies used only phosphate buffered saline (DBS size ranging from 1.1-mm to 12-mm diameter, quantity of buffer ranging from 100 μl to 450 μl) to elute the DBS [17–19,25,26,32,35,37,44]. Eight studies used phosphate buffered saline with Tween (DBS size ranging from 3-mm to 12-mm diameter, quantity of buffer ranging from 50 μl to 800 μl) to elute the DBS [17,20,29,30,39,40,42,52]. Two studies compared the effect of different types of elution buffers [17,46]. Villar *et al*’s study found that DBS samples eluted in PBS/0.5% BSA had the lowest levels of non-specific reactivity in comparison to PBS alone, PBS/Tween 20 0.05%, PBS/Tween 20 0.05%/0.005% Sodium azide and PBS/Tween 20 0.2%/5% BSA. Whereas, Ma *et al* found that eluting DBS spots in 500 μl of 1%Tween-20/PBS resulted in the highest antibody recovery.

### Diagnostic performance

Thirty of the studies used enzyme-linked immunosorbent assays (ELISA) [14–17,19,21–26,29–32,34,36,38,39,41–47,49–52] with the one study using the Luminex 100 [37] or a combination of Treponema pallidum particle agglutination assay (TPPA), Treponema pallidum hemagglutination assay (TPHA) and ELISA [40]. Two studies used the architect system [27,35] as a detection method and two studies used chemiluminescence immunoassay (CIA) either alone [18] or in combination with ELISA [20]. The method of antibody quantification was unclear in one study [35] and not reported in another study [28] (Table 2).

**Fig 2:**
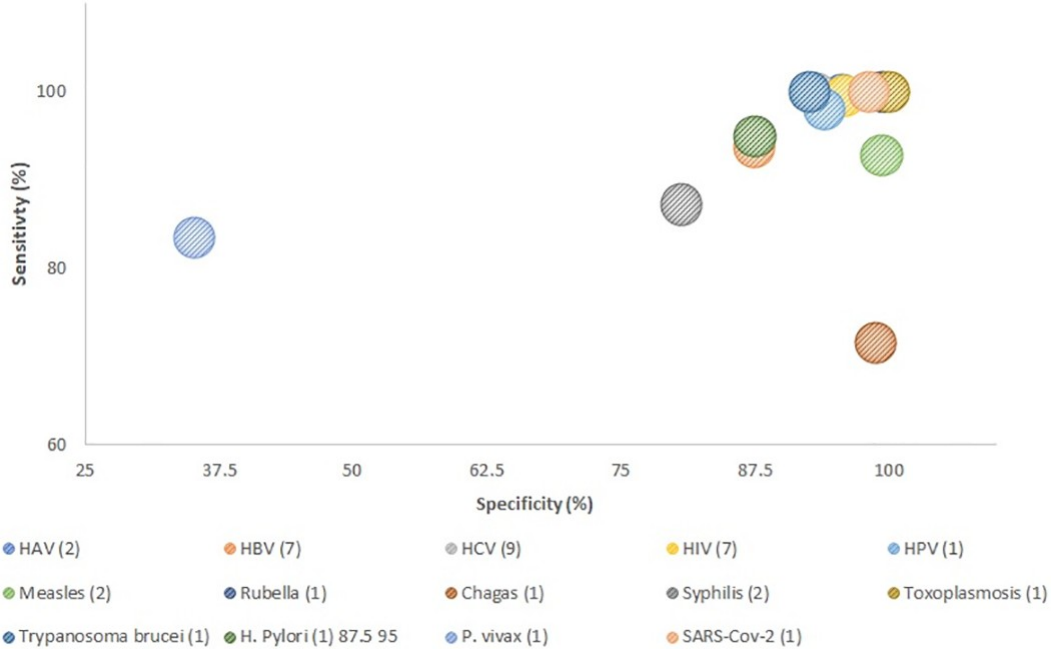
Scatterplot of diagnostic performances (sensitivity and specificity) based on pathogen type.

Thirty-three of the included studies reported the sensitivity [14–28,30–35,37,40,42,44–52] and twenty-six reported the specificity [14,15,17–28,30–33,35,40,44–49,51,52] of antibodies on DBS (Fig 2). The pooled sensitivity for all the infectious diseases ranged from 35.2% to 100% with a mean of 98.8% and the pooled specificity ranged from 50.4% to 100% with a mean of 95.4%. The highest mean sensitivity and specificity reported were for HIV; 97.5% and 99.6%, respectively [23–28].

The lowest mean sensitivity reported were for the malaria (*P*. *vivax*) study; 50% [49] whereas the lowest specificity reported were for syphilis; 50.4% (ELISA) [40] (Table 3).

**Table 3:** Sensitivity, specificity and *r*^*2*^ of the included studies.

AUTHOR, YEAR	SENSITIVITY	SPECIFICITY	CORRELATION (*R*^*2*^) OF ANTIBODY LEVELS IN DBS AND SERUM/PLASMA
**GIL A, 1997** [14]	91.30%	99.30%	Nr
**MELGACO J, 2011** [15]	100%	100%	0.6262
**FLORES L, 2018** [16]	Anti-HBs in HIV- individuals: 79.8% Anti-HBs in HIV+ individuals: 76.8%	Nr	Nr
**VILLAR M, 2011** [17]	HBsAg: 95.45%; Anti-HBc: 89.19%; Anti-HBs: 83.05%	HBsAg: 70.79%; Anti-HBc: 97.53%; Anti-HBs: 81.33%	0.832
**MOHAMED S, 2013** [18]	98%	100%	0.98
**DOKUBO K, 2014** [19]	Anti-HCV: 70%	Anti-HCV: 100%	0.69
**TEJADA-STROP A, 2015** [20]	CIA- 100%; ELISA- 97%	CIA- 100%; ELISA- 100%	Nr
**TUAILLON E, 2010** [21]	99%	98%	Nr
**BRANDAO C, 2013** [22]	95.00%	100%	0.971
**SARGE-NIJE R, 2006** [23]	Ranged from 95% to 100% [depending on which assay was used]	Ranged from 97.5% to 100% [depending on which assay was used]	Nr
**CASTRO A, 2008** [24]	100%	99.60%	Nr
**BOILLOT F, 1997** [25]	87.50%	100%	Nr
**KANIA D, 2013** [26]	HBsAg: 96%; Anti-HBc: 99.3%; HIV: 100%; HCV: 100%	HBsAg: 100%; Anti-HBc: 98.7%; HIV: 100%; HCV: 100%	HBsAg: 0.98; Anti-HBc: 0.98; HIV: 1.00; HCV: 1.00
**ROSS S, 2013** [27]	HBsAg: 98.6%; Anti-HBc: 97.1%; Anti-HBs: 97.5%; Anti-HCV: 97.8%; Anti-HIV ½: 100%	HBsAg: 100%; Anti-HBc: 100%; Anti-HBs: 100%; Anti-HCV: 100%; Anti-HIV ½: 100%	Nr
**LEE C, 2011** [28]	HIV: 100%; HBsAg: 96.5%; Anti-HBs: 74.2%; Anti-HCV: 100%	HIV: 100%; HBsAg: 97.8%; Anti-HBs: 86.9%; Anti-HCV: 100%	HIV Ag/Ab: 0.824; HBsAg: 0.432; Anti-HBs: 0.721; Anti-HCV: 0.631
**BHATIA R, 2019** [29]	Nr	Nr	Nr
**LOUIE K, 2018** [30]	94%	98%	0.961
**UZICANIN A, 2011** [31]	Measles (week 1 after rash): 98.7% Non-measles: 50%	Measles (week 1 after rash): 88.9% Non-measles: 100%	Measles (week 1 after rash): 0.71 Non-measles: 0.33
**COLSON K, 2015** [32]	100%	96.8%	0.92
**PUNNARUGSA V, 1991** [33]	99.40%	100%	Nr
**HELFAND F, 2007** [34]	IgM: 82% IgG: 89%	Nr	IgM: 0.91 IgG: 0.94
**HOLGUIN A, 2013** [35]	98.80%	71.60%	0.803
**EICK G, 2017** [36]	Nr	Nr	0.93
**WATERBOER T, 2012** [37]	HPV: 94.5%; *H*. *pylori*: 96.6%; HCV: 98%; Polyomavirus: 98%	Nr	*H*. *pylori*, HCV, JCV: 0.88; HPV: 0.79
**DUARTE E, 2002** [38]	Nr	Nr	0.95
**FORMENTI F, 2016** [39]	Nr	Nr	0.921
**SMIT P, 2013** [40]	TPPA: 85.4% TPHA: 50.5% ELISA: 94.6%	TPPA: 98.9% TPHA: 99.7% ELISA: 50.4%	Nr
**VAN OMMEN C, 2012** [41]	Nr	Nr	0.91
**CONDORELLI F, 1994** [42]	Measles: 98.6%; Mumps: 96%; Rubella: 99.1%	Nr	Nr
**AABYE M, 2012** [43]	Nr	Nr	Nr
**HEGAZY M, 2020** [44]	100%	100%	Nr
**GEERTS M, 2020** [45]	92.6%	100%	Nr
**MA J, 2020** [46]	Anti-HCV: 98.32% Anti-HIV: 88.46% Anti-TP: 92.19%	Anti-HCV: 100% Anti-HIV: 100% Anti-TP: 100%	Anti-HCV: 0.99 Anti-HIV: 0.96 Anti-TP: 0.95
**KUMAR A**, [47]	Anti-*H*. *pylori*: 87.5%	Anti-*H*. *pylori*: 95%	Nr
**VILLAR L, 2020** [48]	HBsAg: 100%; Anti-HBc: 66.6%; Anti-HCV: 75%	HBsAg: 98.9%; Anti-HBc: 99.8%; Anti-HCV: 99.8%	Nr
**ROSAS-AGUIRRE A, 2020** [49]	35.2%	83.5%	Nr
**STEFIC K, 2019** [50]	98.9%	Nr	Nr
**CRUZ H, 2020** [51]	HBsAg: 81.2% Anti-HBc: 66.5% Anti-HBs: 60%	HBsAg: 93.5% Anti-HBc: 99.3% Anti-HBs: 75.1%	Nr
**MORLEY G, 2020** [52]	98.11%	100%	0.975

Abbreviations: ^a^ anti-HBc: Hepatitis B core antibody; ^b^ anti-HBs: Hepatitis B surface antibody; ^c^ CIA: Chemiluminescence immunoassay; ^d^ ELISA: Enzyme-linked immunosorbent assay; ^e^ HAV: Hepatitis A virus; ^f^ HCV: Hepatitis C virus; ^g^ HIV: Human Immunodeficiency virus; ^h^ HPV: Human papillomavirus; ^i^ HBsAg: Hepatitis B surface antigen ^j^ Nr: Not reported; ^k^ TPPA: Treponema pallidum particle agglutination assay; ^l^ TPHA: Treponema pallidum hemagglutination assay.

Nineteen studies reported the correlation between antibody concentration in DBS and serum or plasma samples [13,15–17,20,24,26,28–30,32–37,39]. The pooled correlation of antibody concentration in DBS and serum/plasma samples ranged from 0.43 to 1.00 with a mean of 0.86. The highest correlation of antibody titers in DBS and serum/plasma samples was observed for Coronavirus, which was 0.97 [52]. The lowest mean correlation of DBS and serum/plasma were observed for the measles study; 0.33 [31] (Table 3).

### Storage of DBS cards

Twenty-one studies reported that their DBS samples were stored at -20°C [17,19,22–24,26,27,30,33,34,36,39,41–43,46,48–50,53], three studies stored at -80°C [37,38,44], five studies stored at 4°C [16,31,32,44,51], two studies stored at room temperature [20,35] and in two studies the DBS’s were stored at -5°C to -10°C [40] and -70°C [21], respectively (Table 2).

Thirteen studies investigated the stability of antibodies in DBS samples stored at different temperatures (Table 4). Six studies [2,21,23,35,55] concluded that antibody levels in DBS were stable at room temperature, ranging from 7 days to 28 days. A slight decline was observed in antibody concentrations in DBS samples that were stored at 2–8°C, although five studies [2,26,46,55,58] demonstrated that DBS samples were stable for up to 210 days at this temperature (range of storage time: 7 to 210 days). Five studies [26,38,55,57,58] found that antibodies in DBS samples stored at 37°C were unstable with antibody concentrations steadily declining in as few as three days [55]. One study showed that antibodies in DBS samples stored at 37°C were stable until the 7^th^ day [2]. Nine studies [2,19,24,26,38,46,55–57] demonstrated minimal variations in antibody concentrations compared to baseline cards stored at -20°C, over 21 to 200 days.

**Table 4:** Optimal temperature for dried blood spot storage.

AUTHOR, YEAR	TEMPERATURE	DAYS	OPTIMUM TEMPERATURE TO STORE DBS FOR MAXIMUM ANTIBODY RECOVERY
**IYER A, 2018** [2]	37°C (humid), 37°C (non-humid), Rmt (~22°C), 4°C, -20°C	7	The vibriocidal antibodies on DBS samples were stable across all temperatures for one week.
**VILLAR M, 2011** [17]	Rmt (22°C to 25°C bag), Rmt (22°C to 25°C), 4°C to 8°C, -20°C	183	For the first 63 days, samples (non-reactive DBS samples to anti-HBc markers) stored in all the temperatures (4°C, -20°C and room temperature) were stable. However, on day 183, samples stored at -20°C had the lowest variation of optical density values. Reactive DBS samples (HBsAg marker) that were stored at -20°C were the only ones which did not result in false-positive. The DBS samples (HBsAg marker) stored at Rmt became ELISA negative on the 63^rd^ day. DBS samples (anti-HBs) stored at Rmt resulted in one false-positive result on the 183^rd^ day. Nevertheless, the DBS samples (anti-HBs) stored at -20°C had the lowest OD variation.
**MOHAMED S, 2013** [19]	Rmt	14	DBS samples (HBsAg and anti-HBs markers) stored at room temperature were stable for up to 14 days.
**TUAILLON E, 2010** [21]	Rmt	12	DBS samples (anti-HCV marker) stored at room temperature were stable for as long as 12 days.
**BRANDAO C, 2013** [22]	Rmt (22°C to 26°C), 2°C to 8°C, -20°C	117	DBS samples (anti-HCV marker) stored at Rmt observed a significant decline in mean optical density on the 117^th^ day in comparison to the baseline. The lowest optical density variation was observed in DBS samples stored at -20°C until day 117^th^ days.
**CASTRO A, 2008** [24]	37°C, Rmt (mean ~22.1°C), 4°C, -20°C, -70°C	42	DBS samples (anti-HIV1 marker) stored at 37°C degraded after the 4th week. The samples stored at Rmt were stable on the 1^st^ week, however, on the sixth week one samples which was HIV-positive (with a low optical density) became HIV-negative. The DBS samples stored at 4°C, -20°C and -70°C were stable until the 6^th^ week.
**PUNNARUGSA V, 1991** [33]	Rmt	28	Antibodies in DBS samples stored at room temperature were stable for as long as 28 days.
**EICK G, 2017** [36]	37°C, Rmt, -20°C	21	DBS stored (EBV markers) at 37°C, room temperature and -20°C were stable for 21 days. Antibodies in DBS samples stored at 37°C decreased over time. Recovery of antibodies in DBS samples stored at -20°C had no clear change; indicating that -20°C is the optimum storage temperature for DBS.
**HEGAZY M, 2020** [44]	4°C, -20°C	4°C: 1 month -20°C: 3 months	Antibodies in DBS samples stored at 4°C were stable for one month and in samples that were stored at -20°C for three months.
**YEL L, 2015** [53]	36°C to 38°C, Rmt, 2°C to 8°C, -20°C to -40°C	14	IgG and IgM antibodies in DBS samples were stable at Rmt, 2°C to 8°C and -20°C to -40°C until the 14^th^ day. However, IgG and IgM antibodies in DBS samples stored at 36°C to 38°C resulted in a decrease of antibody concentration after the 4^th^ day. On the other hand, IgA antibodies in DBS samples stored at Rmt and 2°C to 8°C were stable until the 14^th^ day. The DBS samples (IgA) stored at -20°C to -40°C were only stable until the 10^th^ day, whereas the samples which were stored at 36°C to 38°C were only stable until the 3^rd^ day.
**MARQUES B, 2012** [54]	Rmt (20°C to 26°C), 2°C to 8°C, -20°C	117	DBS samples (anti-HCV marker) stored at 2°C to 8°C and at -20°C were stable until the 117^th^ day. However, the samples (anti-HCV marker) that were stored at Rmt were stable until the 60^th^ day, followed by a reduction in antibody concentration on the 117^th^ day.
**MCALLISTER G, 2015** [55]	37°C, Rmt (22°C to 28°C), 4°C, -20°C, -70°C	200	There was a loss of antibody concentration in DBS samples (HBsAg and anti-HBc markers) stored at 37°C, Rmt and 4°C by the 14^th^ day. The samples that were stored at 37°C resulted in HBsAg negative for all three patients on the 200^th^ day. However, the samples (HBsAg and anti-HBc markers) that were stored at -20°C and at -70°C were stable until the 200^th^ day—as there was minimal variation. DBS samples (anti-HCV marker) were stable at all the temperatures except at 37°C until the 200^th^ day. Samples which were stored at 37°C, resulted in a decline of reactivity by 89% on the 200^th^ day.
**SINGH, P** [56]	37°C, 2°C to 8°C	210	The DBS samples (positive for anti-HEV IgM) that were stored at 37°C were significantly degraded, as 71.43% of the samples became negative on the 55^th^ day, therefore samples were not further analysed. The samples that were stored at 2°C to 8°C were stable when tested on the 65^th^ day as all samples were still anti-HEV IgM positive. However, on the 100^th^ and 130^th^ day, 9.52% and 19.04% of the samples became anti-HEV IgM negative. Aside from this, no IgM fluctuation was observed until the 210^th^ day.

Abbreviations: ^a^ anti-HBc: Hepatitis B core antibody; ^b^ anti-HBs: Hepatitis B surface antibody; ^c^ DBS: Dried blood spot; ^d^ EBV: Epstein-barr virus; ^e^ HBsAg: Hepatitis B surface antigen; ^f^ HCV: Hepatitis C virus; ^g^ HIV: Human immunodeficiency virus; ^h^ IgA: Immunoglobulin A; ^i^ IgG: Immunoglobulin G; ^j^ IgM: Immunoglobulin M; ^k^ OD: Optical density; ^l^ RMT: Room temperature.

### Storage of DBS cards

Twenty-one studies reported that their DBS samples were stored at -20°C [15,17,20–22,24,25,28,31,32,34,37,39–41,44,46–48,51], three studies stored at -80°C [35,36,42], five studies stored at 4°C [14,29,30,42,49], two studies stored at room temperature [18 = 33] and in two studies the DBS’s were stored at -5°C to -10°C [38] and -70°C [19], respectively (Table 4).

Thirteen studies investigated the stability of antibodies in DBS samples stored at different temperatures (Table 4). Six studies [2,19,21,33,54] concluded that antibody levels in DBS were stable at room temperature, ranging from 7 days to 28 days. A slight decline was observed in antibody concentrations in DBS samples that were stored at 2–8°C, although five studies [2,24,44,53,56] demonstrated that DBS samples were stable for up to 210 days at this temperature (range of storage time: 7 to 210 days). Five studies [24,36,53,55,56] found that antibodies in DBS samples stored at 37°C were unstable with antibody concentrations steadily declining in as few as three days [53]. One study showed that antibodies in DBS samples stored at 37°C were stable until the 7^th^ day [2]. Nine studies [2,17,22,24,36,44,53–55] demonstrated minimal variations in antibody concentrations compared to baseline cards stored at -20°C, over 21 to 200 days.

## Discussion

To our knowledge, this is the first comprehensive systematic review to summarise the utility of DBS considering the key aspects of storage, assay methods and card handling which are all important considerations for vaccine trials and serological studies. Overall, the diagnostic accuracy and precision was high when comparing serum/plasma to DBS, indicating that DBS are a useful alternative to serum.

In a review of anti-HCV antibodies eluted from DBS, Vazques-Moron *et al* reported a sensitivity of >96% and a specificity of >99% for anti-HCV antibodies in DBS samples [57]. Their reported figures are similar to the pooled diagnostic performances we have shown. However, our pooled results indicate that there may be differences in both sensitivity and specificity depending on the pathogen type. A study of SARS-COV-2 antibodies demonstrated that sensitivity of matched plasma and DBS was 98.9% [52]. This is potentially useful knowledge during the pandemic as DBS could be used as an alternative to blood samples for national surveillance. DBS sampling would be more convenient for sampling as it does not require attendance at clinic to collect samples and samples can be easily sent back by post.

There are no guidelines on how DBS samples should be stored for short- and long-periods of time and this is evident from the variable storage described in the studies we reviewed. We have demonstrated that storage at room temperature (22–28°C) is acceptable for up to 28 days; making the transportation of DBS samples straightforward, especially in environments lacking cold chain. However, we also report that longer term storage should be at refrigerated or frozen temperatures after 28 days at room temperature, as antibodies degraded significantly thereafter. Overall, our results indicate that -20°C is the optimum temperature to store DBS samples for prolonged periods and it may be necessary for this to be factored into trials where samples may be stored for several years prior to use. This is consistent with the national laboratory guidelines in Denmark [58], Scotland [59], US [60] and Germany [61], which all recommend long term freezer storage. Williams *et al* [62] re-quantified anti-HIV antibodies in four high positive controls that were initially spotted 23 years prior to the analysis. The antibody concentrations obtained were the same as those measured 23 years prior when stored at -20°C. Yel *et al’s* [44] study found that IgA antibodies were stable for up to 14 days at room temperature and at 4–8°C, however, IgA antibodies were stable for up to 10 days when stored at -20°C. Going forward, it may be useful to determine the stability of the different antibody isotypes, as certain antibodies may be more stable than others on DBS.

Whilst this review provides support for the use of DBS for the investigation of immunity to several pathogens, we found only four studies which investigated the antibody concentration against bacterial infections. It is vital that more research is undertaken to understand the stability of antibodies against bacterial infections in DBS samples and how antibody concentrations compare to serum or plasma samples. This is of particular importance if DBS are to be used in vaccine trials against bacterial pathogens, which are required to reduce the impact of the continued spread of antibiotic resistance.

There is of considerable urgency as many bacterial infections are becoming increasingly antibiotic resistant and DBS could be used as part of studies to measure vaccine or natural immunity to bacterial infections [63].

There are several limitations to this systematic review. Firstly, the quality of the studies included in this review were generally low (S1 Table), which precluded a meta-analysis of the data. The filter papers, blood volume collected, size of dried blood spots, elution process and the assays used for antibody quantification also differed amongst the studies, compounding the limited translation of results. Furthermore, the variability of the study designs may have also contributed to the heterogeneity which has restricted direct comparisons and prevented any meta-analysis of data, even of the same pathogen. Secondly, differences in specificity, sensitivity and correlation were noted for different pathogens, suggesting that antibodies against some pathogens in DBS may be less stable than others. Thirdly, the studies did not investigate the effect of humidity on DBS, which is often an issue in sub-Saharan Africa and Asia. Finally, we acknowledge that heterogeneity exists when different cut-off levels are applied between the studies.

Further data are needed to demonstrate the stability of DBS for different pathogens, especially bacteria, under different field transport and storage conditions likely to be encountered in low resource settings, including the effect of high ambient temperature or humidity levels.

Consideration of the use of DBS sampling in clinical vaccine or sero-epidemiological studies will depend on both healthcare setting and available infrastructure. The current lack of guidelines for the adaptation of assays from serum to DBS and on the optimal pre-analytical treatment of specimens makes quality control challenging. With optimal storage, DBS can be a useful adjunct to serological analysis due to their relative simplicity to take and requirements for a less rigorous cold chain, saving time and reducing costs.

## Supporting information

S1 Table Risk of bias in included studies. (DOCX)

S1 Method Search strategy. (DOCX)

S2 Method Prisma checklist for systematic review and meta-analysis. (DOCX)
