# Supplementary material for: Reliability of dried blood spot (DBS) cards in antibody measurement: A systematic review
Source: PLoS One. 2021 Mar 15;16(3):e0248218. doi: 10.1371/journal.pone.0248218 (PMC7959368; doi:10.1371/journal.pone.0248218)
Supplement: S1 Table — (DOCX) [file pone.0248218.s001.docx]

Table S1. Risk of bias for studies using the ROBINS-I assessment

|  | Confounding | Selection of participants | Classification of interventions | Deviations from intended interventions | Missing data | Measurement of outcomes | Reporting bias | Overall bias |
| --- | --- | --- | --- | --- | --- | --- | --- | --- |
| Van Ommen 2012 | Serious risk | Low risk | Low risk | Unclear risk | Unclear risk | Unclear risk | Serious risk | Serious risk |
| Flores 2018 | Serious risk | Low risk | Low risk | Unclear risk | Unclear risk | Unclear risk | Serious risk | Serious risk |
| Villar 2011 | Serious risk | Low risk | Low risk | Unclear risk | Unclear risk | Unclear risk | Moderate risk | Serious risk |
| Aabye 2012 | Serious risk | Low risk | Low risk | Unclear risk | Unclear risk | Unclear risk | Serious risk | Serious risk |
| Kania 2013 | Serious risk | Low risk | Low risk | Unclear risk | Unclear risk | Unclear risk | Serious risk | Serious risk |
| Dokubo 2014 | Serious risk | Low risk | Low risk | Unclear risk | Unclear risk | Unclear risk | Moderate risk | Serious risk |
| Formenti 2016 | Serious risk | Low risk | Low risk | Unclear risk | Unclear risk | Unclear risk | Moderate risk | Serious risk |
| Gil 1997 | Serious risk | Low risk | Low risk | Unclear risk | Unclear risk | Unclear risk | Serious risk | Serious risk |
| Condorelli 1994 | Serious risk | Low risk | Low risk | Unclear risk | Unclear risk | Unclear risk | Serious risk | Serious risk |
| Ross 2013 | Serious risk | Low risk | Low risk | Unclear risk | Unclear risk | Unclear risk | Moderate risk | Serious risk |
| Punnarugsa 1991 | Serious risk | Low risk | Low risk | Unclear risk | Unclear risk | Unclear risk | Serious risk | Serious risk |
| Bhatia 2019 | Serious risk | Low risk | Low risk | Unclear risk | Unclear risk | Unclear risk | Moderate risk | Serious risk |
| Tejada-Strop 2015 | Serious risk | Low risk | Low risk | Unclear risk | Unclear risk | Unclear risk | Serious risk | Serious risk |
| Holguin 2013 | Serious risk | Low risk | Low risk | Unclear risk | Unclear risk | Unclear risk | Moderate risk | Serious risk |
| Mohamed 2013 | Serious risk | Low risk | Low risk | Unclear risk | Unclear risk | Unclear risk | Moderate risk | Serious risk |
| Tuaillon 2009 | Serious risk | Low risk | Low risk | Unclear risk | Unclear risk | Unclear risk | Serious risk | Serious risk |
| Waterboer 2012 | Serious risk | Low risk | Low risk | Unclear risk | Unclear risk | Unclear risk | Moderate risk | Serious risk |
| Uzicanin 2011 | Serious risk | Low risk | Low risk | Unclear risk | Unclear risk | Unclear risk | Moderate risk | Serious risk |
| Helfand 2007 | Serious risk | Low risk | Low risk | Unclear risk | Unclear risk | Unclear risk | Serious risk | Serious risk |
| Louie 2018 | Serious risk | Low risk | Low risk | Unclear risk | Unclear risk | Unclear risk | Moderate risk | Serious risk |
| Lee 2011 | Serious risk | Low risk | Low risk | Unclear risk | Unclear risk | Unclear risk | Moderate risk | Serious risk |
| Sarge-Nije 2006 | Serious risk | Low risk | Low risk | Unclear risk | Unclear risk | Unclear risk | Serious risk | Serious risk |
| Castro 2008 | Serious risk | Low risk | Low risk | Unclear risk | Unclear risk | Unclear risk | Moderate risk | Serious risk |
| Duarte 2002 | Serious risk | Low risk | Low risk | Unclear risk | Unclear risk | Unclear risk | Serious risk | Serious risk |
| Boillot 1997 | Serious risk | Low risk | Low risk | Unclear risk | Unclear risk | Unclear risk | Serious risk | Serious risk |
| Brandao 2013 | Serious risk | Low risk | Low risk | Unclear risk | Unclear risk | Unclear risk | Moderate risk | Serious risk |
| Smit 2013 | Serious risk | Low risk | Low risk | Unclear risk | Unclear risk | Unclear risk | Moderate risk | Serious risk |
| Melgaco 2011 | Serious risk | Low risk | Low risk | Unclear risk | Unclear risk | Unclear risk | Moderate risk | Serious risk |
| Colson 2015 | Serious risk | Low risk | Low risk | Unclear risk | Unclear risk | Unclear risk | Moderate risk | Serious risk |
| Eick 2016 | Serious risk | Low risk | Low risk | Unclear risk | Unclear risk | Unclear risk | Serious risk | Serious risk |
| Hegazy 2020 | Serious risk | Low risk | Low risk | Unclear risk | Unclear risk | Unclear risk | Moderate risk | Serious risk |
| Geerts 2020 | Serious risk | Low risk | Low risk | Unclear risk | Unclear risk | Unclear risk | Serious risk | Serious risk |
| Ma 2020 | Serious risk | Low risk | Low risk | Unclear risk | Unclear risk | Unclear risk | Moderate risk | Serious risk |
| Kumar 2019 | Serious risk | Low risk | Low risk | Unclear risk | Unclear risk | Unclear risk | Moderate risk | Serious risk |
| Villar 2020 | Serious risk | Low risk | Low risk | Unclear risk | Unclear risk | Unclear risk | Moderate risk | Serious risk |
| Rosas-Aguirre 2020 | Serious risk | Low risk | Low risk | Unclear risk | Unclear risk | Unclear risk | Serious risk | Serious risk |
| Stefic 2019 | Serious risk | Low risk | Low risk | Unclear risk | Unclear risk | Unclear risk | Serious risk | Serious risk |
| Cruz 2020 | Serious risk | Low risk | Low risk | Unclear risk | Unclear risk | Unclear risk | Moderate risk | Serious risk |
| Morley 2020 | Serious risk | Low risk | Low risk | Unclear risk | Unclear risk | Unclear risk | Moderate risk | Serious risk |
