# Supplementary material for: Reliability of dried blood spot (DBS) cards in antibody measurement: A systematic review
Source: PLoS One. 2021 Mar 15;16(3):e0248218. doi: 10.1371/journal.pone.0248218 (PMC7959368; doi:10.1371/journal.pone.0248218)
Supplement: S1 Method — (DOCX) [file pone.0248218.s002.docx]

**Supplementary Methods 2. Search strategy**

**The following search strategy was used to search EMBASE (OVID® 1974 to 2019 Week 30) from January 1, 1990 to October 15, 2020 [search conducted on October 15, 2020]: Results= 667**

#1 Guthrie card*.mp. [mp=title, abstract, heading word, drug trade name, original title, device manufacturer, drug manufacturer, device trade name, keyword, floating subheading word, candidate term word]

#2 limit 1 to (humans and yr="1990 - 2019")

#3 dried blood spot*.mp. [mp=title, abstract, heading word, drug trade name, original title, device manufacturer, drug manufacturer, device trade name, keyword, floating subheading word, candidate term word]

#4 limit 3 to (humans and yr="1990 - 2019")

#5 antibod*.mp. [mp=title, abstract, heading word, drug trade name, original title, device manufacturer, drug manufacturer, device trade name, keyword, floating subheading word, candidate term word]

#6 limit 5 to (humans and yr="1990 - 2019")

#7 2 OR 4

#8 6 AND 7

***************************

**The following search strategy was used to search MEDLINE (OVID® 1946 to April, 29 2019 and EBSCO) from January 1, 1990 to October 15, 2020 [search conducted on October 15, 2020]: Results= 789**

The search strategy used in EMBASE was used in MEDLINE OVID and MEDLINE EBSCO.

***************************

**The following search strategy was used to search the Cochrane Library from January 1, 1990 to October 15, 2020 [search conducted on October 15, 2020]: Results= 52**

#1 MeSH descriptor: [Guthrie card] explode all trees

#2 "Guthrie card":ti,ab,kw (Word variations have been searched)

#3 MeSH descriptor: [Dried blood spot] explode all trees

#4 "Dried blood spot":ti,ab,kw (Word variations have been searched)

#5 MeSH descriptor: [Antibody] explode all trees

#6 "Antibody":ti,ab,kw (Word variations have been searched)

#8 1 OR 2 OR 3 OR 4

#9 5 OR 6

#10 8 AND 9

***************************
